# Community health workers at the dawn of a new era: 4. Programme financing

**DOI:** 10.1186/s12961-021-00751-9

**Published:** 2021-10-12

**Authors:** Lizah Masis, Angela Gichaga, Tseday Zerayacob, Chunling Lu, Henry B. Perry

**Affiliations:** 1 Financing Alliance for Health, Nairobi, Kenya; 2 Brigham & Women’s Hospital, Harvard Medical School, Boston, MA United States of America; 3 Department of International Health, Health Systems Program, Johns Hopkins Bloomberg School of Public Health, Baltimore, MD United States of America

**Keywords:** Community health workers, Community health worker programmes, Community-based primary healthcare, Primary healthcare, Universal Health Coverage, Sustainable Development Goals

## Abstract

**Background:**

This is the fourth of our 11-paper supplement on “Community Health Workers at the Dawn of New Era”. Here, we first make the case for investing in health programmes, second for investing in human resources for health, third for investing in primary healthcare (PHC) workers, and finally for investing in community health workers (CHWs).

**Methods:**

Searches of peer-reviewed journals and the grey literature were conducted with a focus on community health programme financing. The literature search was supplemented with a search of the grey literature for information about national health sector plans, community health strategies/policies, and costing information from databases of various countries’ ministries of health, and finally a request for information from in-country partners.

**Results:**

The global shortage of human resources for health is projected to rise to 18 million health workers by 2030, with more acute shortages in Africa and South Asia. CHWs have an important role to play in mitigating this shortage because of their effectiveness (when properly trained and supported) and the feasibility of their deployment. Data are limited on the costs of current CHW programmes and how they compare to government and donor expenditures for PHC and for health services more broadly. However, available data from 10 countries in Africa indicate that the median per capita cost of CHW programmes is US$ 4.77 per year and US$ 2574 per CHW, and the median monthly salary of CHWs in these same countries is US$ 35 per month. For a subset of these countries for which spending for PHC is available, governments and donors spend 7.7 times more on PHC than on CHW programming, and 15.4 times more on all health expenditures. Even though donor funding for CHW programmes is a tiny portion of health-related donor support, most countries rely on donor support for financing their CHW programmes.

**Conclusion:**

The financing of national CHW programmes has been a critical element that has not received sufficient emphasis in the academic literature on CHW programmes. Increasing domestic government funding for CHW programmes is a priority. In order to ensure growth in funding for CHW programmes, it will be important to measure CHW programme expenditures and their relationship to expenditures for PHC and for all health-related expenditures.

**Table:** 

Key message box 1. Summary	
Key findings	
Community health workers (CHWs) are a cost-effective way to extend health services to hardest-to-reach communities. Strong integrated community-based primary healthcare (PHC) systems are essential to accelerate progress toward global goals and to prevent and respond to future pandemics. Investing in CHWs can lead to short-term and long-term cost savings in the health system and help achieve broader societal goals such as women and youth empowerment. Investing in community health yields a 10:1 return on investment. Despite compelling evidence of their effectiveness, CHW programmes are inadequately funded. The lack of national political support and domestic funding for national CHW programmes is perhaps the most critical challenge facing these programmes. Sixty percent of funding for CHW programmes in sub-Saharan Africa is from donors, and most of this is for vertical disease-specific programmes. Determining CHW programme costs and funding requirements is critical for strengthening and expanding these programmes. Mobilizing political will is a prerequisite for moving forward with stronger financing for CHW programmes.	
Key implications	
Long-term financing of CHW programmes is critically important for community health programmes to reach their full potential. Making a compelling case based on the expected return on investment both for improving population health and for promoting socioeconomic development will be critical for generating the political will to ensure long-term domestic funding for CHW programmes. Countries need to be proactive in obtaining alternative sustainable financing for CHW programmes in addition to the existing traditional funding from donors and domestic resources.	

## Background

The financing of national CHW programmes has been a critical element that has not received sufficient emphasis in the academic literature on CHW programmes. At the time the CHW reference guide (*Developing and Strengthening Community Health Worker Programs at Scale: A Reference Guide and Case Studies for Program Managers and Policy Makers* [1]) was published in 2014, it was readily apparent that information about the financing and the costs of CHW programmes was extremely limited, both in the peer-reviewed literature and in the grey literature [2]. Here we provide an expansion of the contents of that chapter by (1) building the argument for why national CHW programmes are a sound financial investment, (2) highlighting the human resource needs in low- and middle-income countries (LMICs), (3) current challenges in financing CHW programmes, and (4) recommendations for strengthening the financing of CHW programmes.

## Why financing PHC and community health programmes should be a high priority

**Table:** 

**Key message box 2**	
CHWs are crucial in strengthening PHC systems. When supported appropriately by other health professionals, the work of CHWs can help to accelerate improvements in the health of underserved populations. CHW programmes are also key for achieving Universal Health Coverage (UHC) and other global goals such as the health-related Sustainable Development Goals	

### The importance of health

We take this for granted, but the importance of health to everyone everywhere bears repeating. The fact that good health is a priority for people throughout the world cannot be overstressed. Health, like education, is among the basic capabilities that give value to human life and that create human capital, one of society’s basic building blocks [3]. Health is routinely put at the top of the list of individuals’ priorities for their own well-being.

Furthermore, access to healthcare is a human right. The Universal Declaration of Human Rights [4], adopted by the General Assembly of the United Nations in 1948, resoundingly affirms that “Everyone has the right to life, liberty and security of person,” that “Everyone has the right to a standard of living adequate for the health and well-being of himself and his family, … including medical care,” and “Motherhood and childhood are entitled to special care and assistance.” We would argue that in today’s context, the “right to life” includes the universal right to accessible basic health-care services that are effective for preventing and treating serious health conditions.

### The broader benefits to developing countries of investing in improving health

There is a renewed awareness of the beneficial effects of investing in health beyond the value that individuals place on health itself and in particular for its effect on stimulating economic growth. Healthier people are more productive. Healthier children are more likely to attend school and have greater cognitive capacity for learning. Improved education is a powerful mechanism of income growth. Good health is the basis for the capability to grow intellectually, physically, and emotionally. Good population health is the foundation for poverty reduction, economic growth, and long-term economic development. Increased life expectancy is an incentive to save for retirement, which can expand the national savings rate, which in turn can expand investment and economic growth. Control of endemic diseases such as malaria and river blindness can increase human access to land and other natural resources [5].

In 2000, WHO’s Commission on Macroeconomics and Health [6], chaired by Jeffrey Sachs, released a seminal report, forcefully making the case that improving the health and longevity of the poor in low-income countries and in lower-middle-income countries is not only important for that benefit alone but also important for poverty reduction and long-term economic growth because, among other reasons, a healthier population has a more productive workforce. Communicable diseases such as malaria and HIV that affect large numbers of people coupled with rising non-communicable diseases and health emergencies produce a drain on economic development. The report highlighted that the interventions required to improve health can be delivered in a “close-to-client” system that requires a “foundation of strong community-level oversight and action, in order to be responsive to the poor, in order to build accountability of local services, and in order to help ensure that families take full advantage of the services provided” [6]. As the WHO Director-General, Tedros Adhanom Ghebreyesus, has recently argued, “Ultimately, primary health care is an investment in a healthier, safer, fairer and more sustainable future” [7].

## The value of investing in community-based PHC services provided by CHWs

There has been strong progress in expanding the evidence that community-based service provision by CHWs supported by other health professionals is effective in improving the health of underserved populations by expanding access to key healthcare services and promoting healthy behaviours.

The deaths of 2.6 million children could be averted each year by expanding the coverage of evidence-based interventions that CHWs can provide [8]. The interventions provided by CHWs that would save the greatest number of lives are (in decreasing order of number of lives saved):

- Immunization of children against pneumococcus, a common cause of childhood pneumonia (this vaccine is now being introduced in many African countries)
- Treatment of diarrhoea with oral rehydration solution and zinc
- Oral antibiotic treatment of childhood malaria
- Oral antibiotic treatment of childhood pneumonia
- Insecticide-treated bed nets and indoor residual spraying (against malaria)
- Thermal care of the newborn (to prevent hypothermia)
- Resuscitation of newborns with asphyxia
- Clean postnatal practices
- Oral antibiotics for neonates with sepsis
- Breastfeeding promotion (especially immediate breastfeeding after birth and exclusive breastfeeding during the first 6 months of life) [8]

CHWs have proved to be a cost-effective way to extend health services to the hardest-to-reach communities [9, 10]. When well integrated within country development agendas and national health strategies, CHWs serve as an entry point to, and interface with, the broader health system for many. Lack of funding and lack of supplies and medicines have been major impediments to the effectiveness of current CHW programmes, as highlighted in the concluding paper of this series [11].

CHWs are increasingly being recognized as a crucial building block in strengthening PHC systems. CHWs are on the front lines of surveillance against emerging infectious threats like COVID-19 and Ebola [12]. They are well positioned to engage communities in preventive and promotive health activities, and to support home-based management of the growing burden of chronic diseases [13]. The evidence shows that programmes using outreach workers that visit homes and provide preventive and curative services in these homes are effective in rapidly increasing coverage of key services and reducing mortality in neonates and children [14], and home-based delivery of family planning services by community-level workers is one of the most effective ways of meeting the unmet demand for contraception [15].

There is now broad awareness of the reality that strong progress in reducing readily preventable and treatable deaths as well as the achievement of UHC cannot be attained in most countries without stronger and expanded CHW programming. The global shortage of human resources for health is one of the reasons for reaching this conclusion, as we discuss below. But also, the effectiveness of CHWs in providing family planning services and diagnosis and treatment of the major causes of death among children (pneumonia, diarrhoea, malaria, and undernutrition) is well established, as we have shown. The ready local availability of CHWs, in contrast to the geographical challenges still present in so many LMIC settings in accessing higher-level workers who are based in facilities, also contributes to the recognition of the need for expanding and strengthening CHW programmes.

The growing consensus now is that services provided by CHWs, when implemented properly, “form the foundation of PHC services by being the first provider sought by families in times of need” [16] (p. 54). The chapter “Community Platforms for Public Health Interventions” in the third edition of the publication *Disease Control Priorities* [17] puts it this way:

Without initiatives to help community platforms flourish around the world, the health gains promised by interventions will cost more and deliver less. Communities will miss opportunities to activate partners and resources that can shift health determinants… (p. 280)

We are now 5 years into the implementation of the global agenda for achieving the Sustainable Development Goals (SDGs), including UHC by 2020. Stronger integrated community-based PHC systems will be essential in order to accelerate progress toward these goals, to prevent and respond to future pandemics, and to confront the dual burden of communicable and noncommunicable diseases. particularly in LMICs but also in underserved areas of high-income countries.

## The value of investing in human resources for health

**Table:** 

**Key message box 3**	
The global shortage of human resources for health is projected to be 18 million health workers by 2030, and the more acute shortages are in Africa and South Asia. CHWs have an important role to play in mitigating this shortage because of their effectiveness (when properly trained and supported) and the feasibility of their deployment	

The *World Health Report 2006: Working Together for Health* [18] brought unprecedented attention to the importance of human resources for health, the heart of each and every health system, emphasizing that progress of low-income countries in expanding immunization coverage, increasing the outreach of PHC, and reducing infant, child, and maternal mortality are all strongly correlated with the density of health workers in the population and with a threshold workforce density below which high coverage of essential interventions will be very difficult. Health programmes and health services cannot be effective without adequate numbers of health staff who are appropriately trained and supported—and who are recruited and deployed according to needs, properly supervised, and work in safe environments.

Investing in health workers of all types is good for economic growth. But investing in health workers who are among the poorest segments of society, particularly those who are women, is particularly productive for economic growth, not to mention the benefits for health and women’s empowerment [5]. CHWs can be trained and deployed much more quickly than can nurses, clinical associates, and doctors, and the cost of CHWs over 25 years of employment is approximately 22% that of a nurse, 15% that of a clinical associate, and 7% that of a doctor (based on average health worker costs across nine East and Southern Africa countries) [19]. Furthermore, many higher-trained health workers have the capacity to migrate to other countries. CHWs would not be doing this [19].

## The global shortage of human resources for health and the potential of CHWs to alleviate this shortage

WHO, in its 2016 report *Global Strategy on Human Resources for Health: Workforce 2030* [20], estimated that in 2013 there was a needs-based shortage of 6.9 million health workers in South-East Asia and 4.2 million in Africa and that this shortage is likely to decline by only 17% by 2030 based on current projections. And, in fact, in Africa, the needs-based shortage will worsen, from 4.2 million to 6.1 million (p. 44). The report also concluded that the aggregate projected global deficit of health workers against needs could exceed 18 million workers by 2030 (p. 46). Further exacerbating the shortage is the maldistribution of the health workforce together with chronic absenteeism, high rates of turnover, and unfilled positions (that are worse in rural areas [21]), leading to situations in which staffing levels are inversely related to levels of poverty and need [22].

Even though the absolute shortage of health workers (in terms of total number) is greatest in South-East Asia, sub-Saharan Africa has the most acute shortage (in terms of numbers of health workers needed per 10 000 population) [20, 23]. In 2006, 57 WHO Member States had a density of health workers below the benchmark of 22.8 doctors, nurses, and midwives per 10 000 population, and in 2018, only 11 of these had progressed to an adequate density of health workers [18, 24]. WHO has estimated that 44.5 basic health workers per 10 000 population will be required to reach the SDGs, but only half of the WHO Member States have the level at present [20]. As we argue below, expanding the numbers and functions of CHWs is one important response to this crisis.

**Table:** 

**Key message box 4**	
Investing in community health programmes yields a 10:1 return on investment. Despite the evidence, domestic spending on health is skewed towards funding tertiary-level care and less for PHC and CHW programmes. Furthermore, funding for CHWs at present is mainly for disease-specific, verticalized programmes. In sub-Saharan Africa, an annual investment gap of US$ 2 billion currently exists for scaling up CHW programmes	

## Methods

A selective search of peer-reviewed journals and grey literature was conducted with a focus on community health programme financing. The literature search was supplemented with a grey literature search for national health sector plans, community health strategies/policies, and costing information from databases of various countries’ ministries of health and in-country partners. The literature that we have been able to draw on was obtained by the authors primarily on the basis of their extensive experience by working in this field and recommendations from colleagues. We did carry out a PubMed search on 12 March 2021 using the terms “financing” and “community health programs”, yielding 1669 articles. Two additional articles that were of relevance [25, 26], but even these did not really address the larger issues of financing large-scale CHW programmes and the value of investing in them.

One of the authors (HP) had recently completed the editing of a book containing 29 case studies of national CHW programmes [27], and each of the case studies had a section on financing. This information, much of which contained unpublished information provided by in-country partners, was helpful for framing our paper. Our findings were also informed by a recent comprehensive assessment of donor spending between 2007 and 2017 [28].

## Financing issues related to CHW programmes

In 2015, a seminal report that led to the formation of the Financing Alliance for Health (FAH) titled *Strengthening Primary Health Care through Community Health Workers: Investment Case and Financing Recommendations* [9] made a powerful economic and impact case for investing in community health, outlined principles for building strong community health platforms, and presented a pathway to sustainably finance those platforms. It found a 10:1 return on investment in community health programmes when accounting for averted mortality, avoidance of high costs of health crises, and the economic impact of increased employment. Furthermore, it found that investing in CHWs can lead to short-term and long-term cost savings in the health system and can help achieve broader societal goals such as women and youth empowerment. However, building strong PHC systems requires adequate investment, across all system components, including investing in human resources for health—one of the most productive ways to invest in health.

Health benefits include not only lives saved and morbidity alleviated, but improved nutrition and the benefits arising from surveillance and pandemic preparedness and response. Benefits for social development include women’s empowerment. SDG 5 is: “Achieve gender equality and empower all women and girls” [29]. Other socioeconomic benefits include helping to address the high rate of unemployment among young people and to turn the “youth bulge” into a “demographic dividend” [19].

### Lack of funding for PHC programmes in general

The Commission on Macroeconomics and Health made the case for a greatly expanded level of development assistance from donor countries for health programmes in low-income countries. The Commission called for greatly expanded global financing for the control of HIV/AIDS, malaria, and tuberculosis (TB), as well as maternal and child health. The Commission also called for new channels of global assistance, including debt relief. Several important funding channels emerged soon thereafter, including the Global Fund for AIDS, TB, and Malaria; the United States President’s Expanded Program for Emergency AIDS Relief (PEPFAR), and the President’s Malaria Initiative (PMI). Development aid for health soared after 2001, and this enabled a major scale-up of many programmes, most notably *vertical* programmes to fight specific diseases.

A review of the impact of three global health funding initiatives which accounted for two-thirds of external donor support for HIV/AIDS control found that these initiatives distorted the countries’ efforts to strengthen health systems [30]. In one striking example, PEPFAR provided Zambia with US$ 150 million, while the entire budget of the health ministry was only US$ 136 million [31].

As a result of all of these distortions, a coalition of international PHC organizations have established the “30 by 2030 Campaign”, calling for international donors to assign 30% of their vertical, top-down, disease-oriented budgets to strengthening integrated, community-based PHC systems by 2030 [32].

And while funding for vertical programmes has dominated external donor support to countries, “hospital centrism” (a term used in the 2008 WHO annual report, which focused on PHC [33]) has dominated domestic government funding for health services. Accurate data are lacking (as are methods for calculating this [22]), but we do know that in high-income countries, the hospital sector accounts for 38% of total health spending compared to 14% on PHC ([34]. According to one report [35], the interquartile range of government PHC expenditures in 36 low-income and lower-middle-income countries was only US$ 15–60 per capita. The 2019 UN General Assembly resolution 74/2 calls for an additional investment of 1% of each countries’ gross domestic product (GDP) for PHC [36].

The Ebola outbreak of 2014 highlighted the weakness and the lack of resilience of the health systems of West Africa and the potential major economic consequences of epidemic outbreaks that are not brought quickly under control. The governments of Guinea, Liberia, and Sierra Leone lost US$ 3.6 billion per year between 2014 and 2017 as a result of the Ebola outbreak (from loss of trade, closure of borders, cancellation of flights, and decreased investment) [37], not to mention the more than US$ 4.3 billion spent by the global community to contain it [38]. Similarly, the COVID-19 pandemic has shown the lack of readiness in health systems to respond to crises and shocks resulting in huge loss of life and severe impacts on societies and economies [39].

Government support for health throughout LMICs and in sub-Saharan Africa particularly has traditionally been underfunded relative to other government priorities. In 2001, the heads of state of countries in the Africa Union met in Abuja, Nigeria, and jointly committed themselves to devote 15% of their annual budget to improve the health sector [40]. And within the government spending on healthcare, hospitals have had “pride of place” along with salaries for physicians and nurses. Thus, funding for PHC and within PHC for CHWs has languished behind. Interestingly, there has been little tracking until recently of how much of the government healthcare expenditures are for PHC relative to hospitals, and within the expenditures for PHC, what percentage is actually spent on CHW programmes.

Hospital centrism is a powerful but infrequently discussed force. Political elites want their leading hospitals to have the best and the latest, as do medical elites, who have often been leading ministries of health (MOHs). MOHs and national government leaders are under enormous political pressure to raise salaries of doctors and nurses—which are often low and irregularly paid—and the threats of doctors and nurses to strike or their actual strikes (the doctors in Kenya were on strike for 6 months in 2019) force decision-makers to give priority to funding them rather than PHC and CHWs. According to one former minister of health in sub-Saharan African, parliamentarians, even from rural areas, are more interested in better funding for hospitals than for PHC and community health services.

**Fig. 1:**
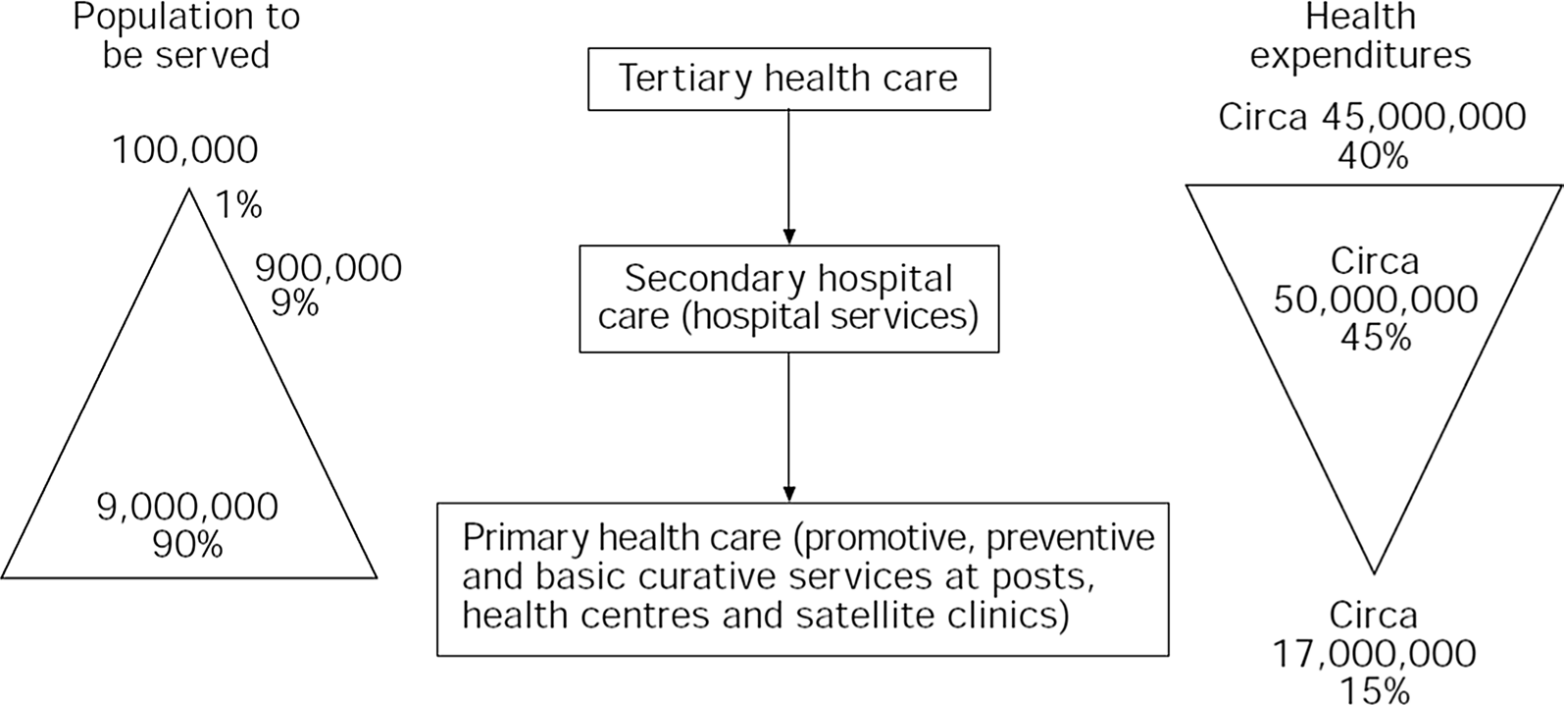
The healthcare dilemma in Ghana, 1978 [37]

Figure 1 provides a startling picture of the scenario for Ghana in 1978. Eighty-five percent of government health expenditures were spent by hospitals and only 15% for PHC.

Perhaps this is an outrageous example because it is outdated and so extreme, but the type of easy-to-understand graphic shown in Fig. 1 should be updated and maintained for all LMIC countries, with the addition of statistics about how much is being spent on CHW programmes. In Uganda, in 2012–13, only 35% of the amount spent by the government for health services was for PHC, while 51% went for hospital services and 14% to other expenditures (mostly management and equipment/supplies) [41].

Spending in the WHO Africa Region on hospitals and specialist care is up to three times higher than the spending on PHC and prevention. In lower-income countries, most of the total spending on PHC comes from donors and from out-of-pocket expenditures. The poorer the country, the lower the share of government health spending goes to PHC. Most donor support for PHC is for vertical programming for specific population subgroups or conditions, with little funding going to support the integrated PHC services needed to achieve UHC [28, 32]. To make matters worse, funding for hospitals disproportionately benefits higher-income groups in the national population [42].

### Lack of funding specifically for CHW programmes

Lack of adequate financing is one of the major stumbling blocks that is keeping national CHW programmes from reaching their full potential [27]. As discussed elsewhere in this series, lack of financing was the most common challenge (tied with lack of supplies) facing 29 recently described national CHW programmes [43]. In fact, in a recent landmark report by the FAH [44] and the United States Agency for International Development (USAID) Center for Accelerating Innovation and Impact (CII) in 2017 established that of the US$ 3.1 billion needed annually for a fully scaled and integrated CHW system in sub-Saharan Africa, only US$ 1.1 billion is currently invested, therefore creating a US$ 2 billion gap [45]. The case for investing to close this gap is compelling, particularly considering the significant human and economic costs of pandemics such as the current COVID-19 pandemic. Current estimates are that the COVID-19 pandemic will likely end up costing between US$ 8.1 and US$ 15.8 trillion globally, more than 500 times the cost of COVID-19 pandemic prevention measures [46].

The 2018 WHO guidelines for CHW programmes [47] emphasize that CHW programmes “require long-term, dedicated financing: attempts to set up and run a large-scale CHW initiative on a shoestring budget is likely to yield disappointing outcomes.” The national Village Health Guides programme of India, launched rapidly in 1977 with inadequate financing and eventually abandoned, is a case in point [48]. And the WHO guidelines for CHW programmes emphasize the need for fair and just remuneration of CHWs [47, 49], which unfortunately is not present in most programmes.

Unfortunately, much of the existing funding supports vertical, disease-specific CHW programmes, despite strong evidence for the efficacy and cost-effectiveness of integrated horizontal platforms [45]. Integrated horizontal programmes offer cost-saving opportunities compared to vertical programmes, which are often duplicative and run in parallel to government health systems. Integrated horizontal programmes can also be more effective, as they build community trust and demand for health services. Yet less than 40% of community health funding today supports the salaried, integrated, horizontal cadres known to be the best investments [45].

The insufficient resources being invested largely come from traditional sources such as donors and domestic governments, which have complex channels, insufficient funding envelopes, and timelines that are, at times, misaligned with country needs. The reality is that donor funds are plateauing and in many cases declining. Domestic funding is stifled by other higher priorities. Sources of domestic financing for CHWs currently exist in some cases, but are as a general rule insufficient. Countries need to be proactive in assembling a financing pathway—a map for the financing mix and transition over time—for their community health programme. This entails obtaining alternative sustainable financing in addition to traditional financing from donors and domestic resources.

MOHs, particularly in sub-Saharan Africa, have frequently made statements to the effect that “we think CHWs are a great idea and will fill a great need but we don’t have any funds to invest in CHWs.” In response, the FAH [44] was established in 2016. The FAH is a multi-organizational partnership that helps governments cost out a well-functioning and effective CHW programme and supports governments on financing strategies for primary care and community health systems that operate at scale that are financially sustainable over time. The FAH partners long-term with MOHs and ministries of finance teams to develop financing pathways relevant to the country’s context and support resource mobilization within the country’s changing financing landscape.

As we anticipate an ever-stronger role for national CHW programmes around the world, we will focus this article on current issues in financing and strategies as well as opportunities for expanding resources that can be used for strengthening these programmes. Due the seriousness of funding issues in sub-Saharan Africa, we will limit our focus to this region of the world. The countries within South Asia and South-East Asia such as India, Bangladesh, and Thailand have programmes that are relatively well funded with sustainable government resources [27].

### Current costs and levels of funding for CHW programmes, and estimated funding shortfalls in sub-Saharan Africa

At present there are at least 637 000 paid CHWS and 3.7 million volunteer CHWs in sub-Saharan Africa working in national CHW programmes that are not part of disease-specific vertical programmes [27, 50]. The number of volunteer CHWs receiving intermittent financial incentives to support vertical programming is unknown but is probably at least 1 million in sub-Saharan Africa [19]. US$ 1.0 billion is being spent on CHW programmes annually in sub-Saharan Africa—about 60% of this from donors and 40% from governments [51].

Sixty percent of the funding for CHW programmes in sub-Saharan Africa is from donors, and most of this is for vertical, disease-specific programmes [28, 45]. A recent study by Lu et al. [28] for the period from 2007 to 2017 estimated that only 2.5% of total health-related development assistance was for CHW programmes, and most of the funding was for vertical disease-control programmes (HIV and other sexually transmitted diseases [STDs]—38.9%, malaria—19.8%, reproductive health—9.3%). Seventy percent of the CHW-directed funds were for countries in sub-Saharan Africa, where the per capita amount of assistance was also the greatest among the various regions of the world (US$ 0.39) during the study period.

As shown in Table 1, information about the costs of CHW programmes in sub-Saharan Africa are available for nine countries (Burkina Faso, Liberia, Malawi, Rwanda, Sierra Leone, South Africa, South Sudan, Zimbabwe, and Zambia) and Zanzibar (a semi-autonomous region of Tanzania). The costs indicated are annual steady-state costs after the programme has been fully deployed. As such, they do not include start-up costs, but are composed of recurring costs once the programme has reached scale. This was done to increase the comparability of the data across countries since start-up costs are widely variable. So the programme costs are pulled for the final year of the strategy, at which point the assumption is that the programmes will be fully scaled up/deployed. All costs have been adjusted to reflect 2019 US dollars. The two different years shown in column 1 of Table 1 refer to the year in which the costs were tabulated, and the second year is the year during which the full, scaled-up programme was implemented or is expected to be implemented. So these costs are to some degree actual (in the case of Rwanda, Sierra Leone, and South Africa) and projected (in the case of the other countries and Zanzibar). But they are the best data currently available.

**Table 1:** Estimates of predicted CHW programme costs for selected countries in sub-Saharan Africa (in US$)

Country	Column 1: Year in which costs were estimated and the final year in which the programme was (or will be) fully deployed	Column 2: Estimated annual cost of CHW programme (in millions), with commodities	Column 3: Estimated annual cost of CHW programme (in millions), without commodities	Column 4: Country population (in millions)	Column 5: CHW programme cost per capita (with commodities)	Column 6: Number of CHWs	Column 7: CHW programme cost per CHW	Column 8: Monthly salary of CHWs	Column 9: Approximate number of people in each CHW’s catchment area
Burkina Faso	2019, 2023	$32. 2	$27.2	20.3	$1.59	13 500	$2 764	$35	1 504
Liberia	2016, 2021	$11.8	$9.5	4.9	$2.41	8 311	$3 115	$70	590
Malawi	2017, 2022	$109.0	$85.7	18.6	$5.86	14 910	$2 384	$135	1 247
Rwanda	2019, 2019	$34.0	$28.1	12.6	$2.70	60 000	$567	$0	210
Sierra Leone	2016, 2020	$30.6	$17.2	7.8	$3.92	13 644	$2 038	$23	572
South Africa	2017, 2017	$743.9	$705.6	58.6	$12.69	95 962	$7 751	$208	611
South Sudan	2019, 2023	$34.1	$27.6	11.1	$3.07	28 755	$1 185	$7	386
Zambia	2018, 2021	$140.2	$68.7	17.9	$7.83	42 140	$3 559	$23	425
Zanzibar	2019, 2025	$2.4	$1.7	1.6	$5.63	2 616	$3 440	$22	612
Zimbabwe	2020, 2025	$51.3	$38.7	7.6	$24.05	17 400	$1 745	$75	437
Median value					$4.77		$2 574	$35*	581

*Does not include Rwanda

All data are reported in 2019 US dollars. These costs are steady-state recurring costs (i.e., the costs of operating CHW programmes after scale-up is complete and the programmes have reached their steady state). It is important to note that not all programmes may reach the steady state due to other bottlenecks such as inadequate financing not being available to complete scale-up.

Calculations: Column 5 = Column 2/Column 4; Column 7 = Column 2/Column 6; Column 9 = Column 4/Column 6

Sources of information: Columns 1–3, 5, 6, 8 [44]; Column 4: [54]

These costs are for national community health programmes largely run by the governments of the respective countries. The United Nations Children’s Fund (UNICEF)/Management Sciences for Health (MSH) Community Health Planning and Costing Tool [52] was used to model scale-up, coverage, and cost of providing community health services over the strategy periods of the respective countries. The Community Health Planning and Costing Tool is a spreadsheet-based tool that helps planners and managers to determine the costs and finances of community health services packages [53]. It allows users to calculate the costs and financing elements linked to all aspects of the CHW package, including service delivery, training, supervision, and management from community to central levels.

Data for the cost of CHW programmes were obtained from various primary sources including review of relevant documents from MOHs such as national health sector plans, community health strategies, from the central statistics offices, and from in-country partners. Literature searches were performed to identify additional information of costs in journal articles and reports in the grey literature. In addition, discussions were usually held with various MOH officials at national and (where relevant) subnational levels and directly with CHW supervisors, CHWs themselves, and partners. Inflation rate and exchange rate data were obtained from the relevant central bank websites. The results of the analysis were validated by a consortium of experts comprising government, partners, and MSH/UNICEF consultants who developed the costing tool. Estimated costs were calculated in two ways—with and without the commodities and medicines that the CHWs would use.^1^

The median estimated cost per person of these CHW programmes is US$ 4.77, ranging from US$ 1.59 in Burkina Faso to US$ 24.05 in Zimbabwe (Table 1, column 5). The median annual programme cost per CHW is US$ 2574, ranging from US$ 567 in Rwanda (where CHWs are volunteers) to US$ 7751 in South Africa, where CHWs earn $208 per month (Table 1, column 7). The median monthly salary of CHWs in the countries that pay their CHWs is $35. The median size of the catchment area for each CHW is 581 people, ranging from 210 in Rwanda to 1504 in Burkina Faso (Table 1, column 9).

Data are available on government spending for PHC for six of the countries in Table 1. Table 2 compares the CHW programme costs for these countries with the government’s expenditures for PHC alone along with its total health expenditures. Since the WHO data on PHC expenditures are through 2017 only, and since the costs for scaled-up CHW programming are projected expenses that have not yet been incurred, we have chosen to compare these two quantities as ratios.

**Table 2:** CHW programme costs and spending on PHC and on all health programming by governments and donors in the sub-Saharan African countries with available data (in 2019 US dollars)

Country	Column 1 Year CHW costs incurred	Column 2 Per capita spending on CHWs^a^	Column 3 Per capita spending on PHC by government and external donors^b^	Column 4 Per capita health spending by domestic government and external donors^b^	Column 5 Ratio of spending on PHC by government and external donors to spending on CHWs	Column 6 Ratio of total health spending by government and external donors to spending on CHWs
Burkina Faso	2019	1.59	33.71	34.82	21.20	21.90
Liberia	2016	2.41	16.94	32.06	7.03	13.30
Rwanda	2019	2.61	42.02^c^	45.68	16.10	17.50
South Africa	2017	12.18	102.67^c^	288.62	8.43	23.70
South Sudan	2019	3.07	13.81	18.22	4.50	5.93
Zambia	2018	7.51	45.53	63.70	6.06	8.48
Median		2.84	37.86	40.25	7.7	15.4

^a^Data source: From Table 1

^b^Data source: https://apps.who.int/nha/database/ViewData/Indicators/en. For health expenditure by governments and donors, WHO only has data available until 2017. We imputed 2018 and 2019 values for government spending on health and donors’ contribution to health using their average growth rates between 2010 and 2017 (i.e., the average growth rate of government health spending, and average growth rate of donors’ contribution). South Sudan has data for 2017 only. We therefore used 2017 data. For PHC expenditure, WHO has data for only 56 countries in 2016 and/or 2017. We assumed the growth rate of PHC spending by government and donors to be the same as the growth rate of health spending by government and donors and imputed PHC spending by government and donors in 2018 or 2019

^c^Data source: https://improvingphc.org/explore-country-data Information on donor spending on PHC was not available for Rwanda and South Africa. We imputed the data for South Africa using (total health aid/total health expenditure) in the country. One study [28] shows that during 2010–2011, about 95% of funds for CHW programmes were from international donors. As available data show that 38% of funding of CHW programme were from governments between 2014 and 2015, we therefore assigned 62% to donors

Estimated CHW programme costs are quite small compared to the government’s current PHC expenditures and, of course, much smaller compared to the government’s total health expenditures. For every dollar spent on CHW programming, the median amount spent by governments on PHC is US$ 7.70 (Table 2, column 5) and on other PHC expenditures is $15.40 (Table 2, column 6). Or, stated alternatively, for every dollar the government is spending on PHC, it is spending 13 cents on CHW programming, and for every dollar the government is spending for all health-related purposes, it is spending 6 cents on PHC programming. Given the importance of CHW programmes for improving population health, as we have emphasized elsewhere in this series of papers, the current funding for these programmes is quite modest and should be able to be expanded significantly with only minimal adjustments to the nation’s health spending. These adjustments could mean substantial increases for the funding of CHW programmes and therefore, assuming these funds are appropriately utilized, in substantial improvements in the effectiveness of these programmes.

### How are national CHW programmes currently funded and is there evidence of transition away from donor dependence?

Rodriguez et al. state, “Overreliance on donor support is often a reflection of limited domestic political commitment” [55]. They go on to say that, as countries transition away from donor funding, “…the building and sustainment of political commitment for health services for vulnerable populations become a critical human rights issue.” Donor dependence also has a hard-to-document perverse effect as well. When governments know that donor support is available for a given type of programme, they have an incentive to divert their own domestic funding to other priorities.

China, Brazil, and India are examples of countries that have financed their CHWs with domestic resources. Chinese barefoot doctors were funded with locally generated revenues from the collective cooperative economy (when there was no private ownership of land). There are few examples of successful programmes that rely primarily on local financial support. A 1983 report indicated that there are numerous examples of failed programmes that depended on local financial support [56]. Fee for service is generally not recommended because it is open to abuse [57].

In Brazil, national, state, and municipal bodies all provide support. These funds go to support the PHC programme as a whole rather than into the CHW programme separately. Decisions over the use of these funds are influenced by civic participation. Councils at the federal, state, and municipal levels address health system issues, including budgets. CHWs are an integral part of the PHC family health teams, and it is the family health team that is funded, not the CHW. Recent government policy has frozen government funding for health, however [58].

India has the largest cadre of paid CHWs in the world, the accredited social health activists (ASHA). They are funded from the central government budget. Since 2006, the Indian Government has budgeted US$ 167 for each ASHA, but the poorest states were unable to absorb these funds [59].

The Bangladesh Rural Advancement Committee (BRAC) CHW programme (in Bangladesh) is also funded with domestic resources—by the CHWs selling health-related commodities for a small profit, providing income to the CHW [60]. The international nongovernmental organization Living Goods adopted this model and promoted its incorporation into government programmes in East Africa.

Anecdotal evidence has supported the notion that government officials in many countries have been fearful of incorporating CHWs as formal MOH employees because of the amount of money that would require as well as because of the likelihood of going on strike for a larger salary. Since MOH funds are so limited, this could be disastrous for MOH programmes. Also, since budget scales are so low, in some countries the entry-level nurse is receiving a minimum wage. If CHWs received a minimum wage, the whole system would have to be revised with substantial cost implications (Kate Tulenko, personal communication, 2014). Also, “wage bill” issues limit how much MOHs can pay CHWs. These are restrictions that are imposed by the World Bank on the percentage of government expenses that can be devoted to salaries. Some of these constraints may be relaxed as part of the COVID-19 global pandemic response.

Kenya, South Africa, and Nigeria are examples of countries with weak financial support for CHW programming. Kenya recently diverted CHW funds to other activities. It has allocated only 2% of UHC funds for CHW programming. However, some counties in Kenya are taxing their constituents to provide funds to pay their volunteer CHWs a small salary [61]. South Africa and Nigeria have both approved plans for funding CHWs, but then never authorized the actual expenditure of these funds [62, 63]. Scaling up South Africa’s CHW programme strategy would cost only 3% of the total public sector health expenditure. An assessment carried out in two districts in South Africa revealed that only 4% of PHC expenditures are currently being used for CHWs [63].

Prior to 2011, Zambia was not devoting any of its domestic resources to a CHW programme. Donor support made it possible to plan and initiate a national CHW programme. In 2011–12, the CHW programme cost US$ 1.8 million, 88% of which was provided by donors. In 2016–2017, total programme support had grown to US$ 8.9 million, with 81% from domestic government resources and only 19% from donors [45]. Liberia, Sierra Leone, Rwanda, and Kenya, among others, are investing their own domestic resources to community health services. An important statistic to monitor is whether the amount of domestic resources going to CHW programmes and to PHC is growing at least as rapidly (and hopefully more rapidly) as total health expenditures (a large portion of which is expenditures for hospital services).

### The need for accurate costing of CHW programmes

Planning for CHW programme strengthening and expansion requires an accurate process for determining current costs and what additional funding will be needed. New tools are now available [53], and more will certainly become available. Of particular importance is the need to plan for and ensure adequate funding for supervision, commodity and supply chain costs, as well as for ongoing training needs and career development programmes to maintain a motivated workforce with minimal CHW attrition. Evidence from the peer-reviewed scientific literature regarding these issues is limited [26]. There is a need for better methods to assess costs from a societal perspective rather than just through the lens of the cost to the government. As mentioned earlier, accounting for associated costs as well as intangible costs and broader societal benefits is important.

### Previous and current attempts to expand funding for CHW programmes globally

In 2012, Jeffrey Sachs, at that time Director of the Earth Institute of Columbia University in New York, initiated a campaign to incorporate 1 million salaried CHWs throughout rural sub-Saharan Africa so that there would be one CHW for every 650 people. The One Million Community Health Workers Campaign estimated (in 2012) that this would require US$ 2.3 billion per year (US$ 3584 per CHW and US$ 6.56 per person served and US$ 2.62 per capita) [10]. Unfortunately, the campaign never gained the traction hoped for.

In 2016, the Joint United Nations Programme on HIV/AIDS (UNAIDS) declared that CHWs were the key to ending the HIV/AIDS epidemic, and called for an additional 2 million CHWs to be recruited, including retraining many former CHWs and giving them a set of duties which would include HIV/AIDS-related tasks [19]. This campaign also never gained the hoped-for traction.

Now, in the midst of the COVID-19 pandemic, the Africa Center for Disease Control and Prevention is planning to enlist 1 million CHWs to support contact tracing across the continent [64]. Given the massive funding that is now coming forth to combat the pandemic, it seems quite likely that the funding will finally be available to make this happen. As one recent commentary proclaimed:

Ongoing efforts to leverage CHWs for the COVID-19 response must not be one-offs in the face of an emergency. CHWs must be equipped, trained, and supported for the long term as a crucial human resource for health. [65]

The authors go on to say, given the trillions of dollars now being committed for the COVID-19 global pandemic response:

A comparative US$ 2 billion annual investment to bolster CHWs as a health system strengthening platform for primary care is a drop on the ocean. Now is the time to invest in community health systems in sub-Saharan Africa and avert a greater crisis. [65]

### Why has funding for CHW programmes been so difficult to obtain, when the evidence of their effectiveness is so abundant?

Why this question has not received more attention is troubling. But much broader goals to expand government funding for health have also been difficult to achieve. Why has this been the case? In April 2001, the heads of state of African Union countries met and pledged to set a target of allocating at least 15% of their annual budget to improve the health sector [66]. Progress toward this goal has been disappointing [40]. One of the explanations is that healthcare has been viewed as a cost and not as a human capital investment, definitely negatively influencing domestic resource allocation.

Unfortunately, evidence often does not guide policy or budgets. There is no evidence that investing in hospitals improves the health of a geographically defined population, but the hospitals have strong political support as well as support from medical elites. Curative medicine, medical specialization, and tertiary medical care are all high-status endeavours in the minds of the general public and decision-makers, while PHC, maternal and child health, public health, and community health are all low-status endeavours throughout the world.

Too often, higher-level health workers above CHWs do not fully appreciate or accept the important role played by CHWs or their potential for playing a stronger role. At some deeper psychological level, more highly trained workers resent the fact that CHWs can be taught and authorized to diagnose and treat with medicines after receiving far less training than they themselves received. Sometimes, CHWs are looked down upon because of their low social status (since CHWs often come from lower-income families in their communities) and their lack of formal education. These issues are discussed elsewhere in this series [67, 68].

And, of course, we have to recognize that women and children are a low priority in the broader political agenda, and CHW programmes have been focused on serving them. CHWs have mostly been women, and gender discrimination has probably contributed to the lack of political support for CHW programming.

**Table:** 

Key message box 5	
The evidence on the effectiveness of CHW programmes is compelling. It is thus critical that technical and financial support towards national CHW programmes be backed by strong national political will to ensure the sustainability of these programmes at the country level	

## Recommendations

### Build stronger political commitment for CHW programming

Mobilizing political will is a prerequisite for moving forward with stronger financing for CHW programmes, and champions are needed to create political will. The recent report authored by FAH and USAID CII asserts:

Mobilizing political will is a prerequisite for developing a community health system and an ongoing requirement for sustaining it. Political will, and the continued advocacy needed to build it, is key to harnessing the resources required to close the funding gap. Diverse champions can build support for community health across ministries of health and finance, donors, and local stakeholders. [45] (p. 15)

The development of a robust CHW workforce needs to be a key political priority championed by the head of state, not just by the minister of health [69]. As the leading political body within the continent, the Africa Union, its Member States, and principal decision-making organs have a key role to play in mobilizing this higher level of political will.

### Develop strong investment cases and plans for community health

An investment plan is a document summarizing a community health strategy, associated costs, expected returns on investment, existing resources to support the strategy, and potential additional financing sources and strategies. It is a guiding document that helps the MOH create a vision and set targets for financing, and the sources of financing. Moreover, this plan serves as a document for stakeholders, including donors and the ministry of finance, that demonstrates a credible, executable, and financially sustainable pathway for the community health programme. The investment plan should be linked to the national community health strategy, considering how to increase the financial sustainability of the programme over time, particularly as donor funds continue to reduce. This investment plan may include detailed financing solutions, for example, newer sources such as social impact bonds if they have been identified. Finally, it should be multiyear and must be updated on a regular basis to reflect changes in the environment for policy, funding, and programme operations. Building on the strong case for investment, each country in sub-Saharan Africa needs to analyse its specific economic, social, and health return on investment that is based on the contextualized community health system that will be built.

### Avail a dedicated team and build capacity in the MOH specifically for community health

A functional team with clear roles and responsibilities under the relevant government office will play a critical role in coordination, mobilizing of resources, and implementing activities in line with the community health programmes. Ministry-led coordination among government, partners, and donors, spearheaded by a central directorate and complemented by local structures, can help to eliminate inefficiencies and create strong integrated programmes.

### Position community health as a key pillar of PHC

There is an urgent need to track expenditures for CHW programmes relative to overall health expenditures and other PHC expenditures, as well as a need to promote the idea that CHW programmes should have a privileged place in terms of increases in investment relative to other health expenditures because of their greater impact in saving lives.

Increased funding for CHW programmes can also be achieved through efforts to increase the overall funding for PHC more broadly. The recently released document, *Primary Health Care on the Road to Universal Health Coverage: 2019 Monitoring Report* [70], outlines some approaches. For low-income countries, these include:

- Increasing domestic public funding on health as a whole
- Increasing donor funding on PHC
- Reallocating donor funding

For lower-middle-income countries, the report adds, in addition to the above, reallocation of domestic public funding needs to be considered. This is unfortunately not an option in low-income countries according to the authors. The report also suggests that in low-income countries, donor investments in PHC programmes should be matched by domestic funding for PHC operations—salaries, medicines, and so forth. The report suggests that if external aid for health increases by 0.5% of the GDP and that if 10% of aid is shifted from non-PHC activities to PHC and if government spending increases by 3% of GDP (and the health share of government spending increases by 4%), PHC spending from public sources would increase from 0.9% to 1.9% of GDP by the year 2030 [70].

Increasing domestic government resources through improving the capacity of governments to tax and generate revenues is a joint commitment across Africa and beyond, known as the Addis Ababa Action Agenda. If priority is given to funding for PHC and for CHW programmes through this approach, then significant additional revenues could be generated.

### Integrate CHWs into the broader health system

Policy frameworks need to be reformulated to authorize task shifting, to formalize and elevate the status of CHWs, to promote their professionalization, and to find the funding to make this happen. Integrated, salaried cadres of CHWs (as opposed to CHWs receiving intermittent limited incentives from vertical, disease-control programmes) are the lynchpin of strong community health systems.^2^ A report coauthored by FAH also declared that “[c]hanneling existing community health funds toward strong, well-compensated, integrated cadres can begin to close the financing gap” [45]. This involves providing fair compensation for CHWs, ensuring proper supervision, access to mobile technology, and performance monitoring. Other healthcare workers also need to be trained to address and overcome potential professional resistance, and steps need to be taken to ensure that CHWs have an organizational voice such as doctors, nurses, and other health workers do. Global, national, and regional bodies are needed for this.

### Explore additional funding avenues for community health programmes

New sources of existing government funding need to be sought as well as flexible start-up funds from donors, including from private equity. Social investment bonds need to be considered as a source of medium-term financing when available [45].

The United Nations and WHO have recommended to its Member States an increase in the annual government expenditures for PHC by 1% of the GDP in order to reach the SDGs and UHC [70]. This would represent a 5% increase beyond the current level of spending on health globally. Part of these funds could be used to support CHW programmes.

Other suggestions proposed by UNAIDS [19] for new financial resources include the following:

- Redirect current funding (for example, polio eradication money and funds for neglected-tropical-disease programmes that are winding down)
- European Union Emergency Trust Fund (for addressing the root causes of international migration of health workers)
- Pandemic Emergency Facility of the World Bank Group; as frontline respondents to health emergencies, CHWs are ideal candidates for funding through this fund
- Africa Community Health Workers Bond (UNAIDS is prepared to work with the African Development Bank on creating this for social impact investors that can convert long-term government pledges into immediately available cash)
- Africa Health Investment Fund (UNAIDS working with the Centre for Global Health and Development to launch a private investment fund with the aim of mobilizing US$ 1 billion, including US$ 150 million in grants for CHW programmes)
- Donor innovations that might be possible include (1) the waiving of debt in return for government commitments to strengthen health systems (including investing in CHWs) and (2) channelling donor funding in return for the achievement of certain health results or outcomes that require CHWs
- The development of national health insurance schemes that pay for services provided by CHWs
- The creation of schemes in which voluntary contributions are sought from the private sector—such as mobile telecommunications users paying 1% of their bill toward the support of CHW

### Strengthen public financial management systems to support health sector priorities

An effective public financial management system ensures that funds are used effectively and efficiently to deliver high-value services. This often means directing funds to priority populations, interventions, and services. Predictable funding allows the MOH to be both realistic and ambitious in designing interventions because they will have greater assurances that adequate funding for priorities will be available, including for community health. The current COVID-19 crisis has made clear that the ability to address health system shocks in an efficient manner depends on the strength and flexibility of a country's public financial management system and its health financial management system, as well as how these systems work together. The COVID-19 pandemic is showing the need for reactive systems that can provide medications, protective equipment, and other supplies to hospitals and other facilities quickly and efficiently, as well as to CHWs. The flexibility of funding flows is key to enhancing funding to cover operational costs within public facilities that are responding to the crisis, and/or reallocating them to functions like contact tracing.

## Potential impact of the COVID-19 pandemic on CHW funding

As already mentioned, trillions of dollars are now being mobilized in a frantic attempt to diminish the effects of the COVID-19 pandemic. There is universal recognition of the critical role of CHWs in this effort not only for the short term but also for the long term to provide resilience for the next pandemic and also to fill a void in the provision of healthcare services that only CHWs can fill. If new funding arrives to strengthen their capacities (as is likely), will this be continued after the pandemic ends? Time will tell.

A report by Ballard et al. in 2020 on the importance of CHWs in the COVID-19 response underscores the need to ensure that any short-term boost for CHW funding continues for the longer term:

The investments in the supply chain, compensation, dedicated supervision, continuous training and performance management necessary for rapid community response in a pandemic are the same as those required to achieve universal health coverage and prevent the next epidemic. Strengthening high-quality healthcare delivery systems will save lives, not just during COVID-19, but always. [71]

Finally, there are growing calls for debt relief for low-income countries, such as by the current Prime Minister of Ethiopia, Abiy Ahmed [72]. Conditions for this could include leveraging the funds released for financing CHW programmes. As of 1 May 2020, many developed countries have suspended the obligation for poor countries to service their debt [73].

## Conclusions

While heretofore, CHW programmes have mostly been an underfunded afterthought, there are reasons for cautious optimism about an expansion in funding available for CHW programmes. It should be accepted as a norm that growth in government spending for CHW programmes should at least parallel, but preferably exceed, the rate of growth in all government health spending, and that spending on CHW programmes becomes a much larger share of spending on PHC than is currently the case. The WHO guidelines for CHW programmes concludes with the following statement: “The key determinant of success in securing adequate levels of investment is the political will to prioritize approaches and strategies that are most likely to lead to improved population health outcomes.” The challenge before us is clear: millions of lives are at stake.

## Data Availability

Any articles and other materials cited by the authors are available from the corresponding author.

## Abbreviations

ASHA: Accredited social health activist
CHW: Community health worker
COVID: Coronavirus disease
FAH: Financing Alliance for Health
GDP: Gross domestic product
LMICs: Low- and middle-income countries
MSH: Management Sciences for Health
MOH: Ministry of Health
PEPFAR: President’s Emergency Program for AIDS Relief
PHC: Primary healthcare
PMI: President’s Malaria Initiative
UHC: Universal Health Coverage
UNAIDS: Joint United Nations Programme on HIV/AIDS
UNICEF: United Nations Children’s Fund
US: United States
USAID: United States Agency for International Development

## Footnotes

1 The cost for CHW programmes shown in Table 1 do not include costs incurred by the CHWs themselves (out-of-pocket expenses for carrying out routine activities as well as opportunity costs, which refers to what CHWs could earn if they were engaged in a different activity), costs to the health system of additional healthcare generated by CHW referrals, costs to patients and their families for services provided by CHWs, and the costs of high CHW turnover (e.g., disruption of services, low staff morale, poor quality, and recruitment of replacements).

2 Sub-Saharan Africa has only 158 000 CHWs who are fully salaried employees (compared to the 1 million that are needed).
